# Modern Approaches in Cardiovascular Disease Therapeutics: From Molecular Genetics to Tissue Engineering

**DOI:** 10.3390/bioengineering8110174

**Published:** 2021-11-04

**Authors:** Panagiotis Mallis, Efstathios Michalopoulos, Catherine Stavropoulos-Giokas

**Affiliations:** Hellenic Cord Blood Bank, Biomedical Research Foundation Academy of Athens, 4 Soranou Ephessiou Street, 115 27 Athens, Greece; smichal@bioacademy.gr (E.M.); cstavrop@bioacademy.gr (C.S.-G.)

**Keywords:** cardiovascular disease, tissue engineering, small-diameter vascular grafts, mesenchymal stromal cells, 3D printing, decellularization, macrophages

## Abstract

Cardiovascular disease (CVD) currently represents one of the leading causes of death worldwide. It is estimated that more than 17.9 million people die each year due to CVD manifestations. Often, occlusion or stenosis of the vascular network occurs, either in large- or small-diameter blood vessels. Moreover, the obstruction of small vessels such as the coronary arteries may be related to more pronounced events, which can be life-threatening. The gold standard procedure utilizes the transplantation of secondary vessels or the use of synthetic vascular grafts. However, significant adverse reactions have accompanied the use of the above grafts. Therefore, modern therapeutic strategies must be evaluated for better disease administration. In the context of alternative therapies, advanced tissue-engineering approaches including the decellularization procedure and the 3D additive bioprinting methods, have been proposed. In this way the availability of bioengineered vascular grafts will be increased, covering the great demand that exists globally. In this Special Issue of *Bioengineering*, we tried to highlight the modern approaches which are focused on CVD therapeutics. This issue includes articles related to the efficient development of vascular grafts, 3D printing approaches and suitable atherosclerosis models.

The development of functional vascular grafts, which can be applied in cardiothoracic surgeries, represents currently one of the greatest goals of tissue engineering. In particular, great effort has been made in order to better optimize the manufacturing procedures for the development of small-diameter vascular grafts (SDVGs) Globally, there is a great demand for this type of graft, applied mostly in coronary artery bypass grafting (CABG) [1,2]. Coronary artery obstruction represents one of the common manifestations of CVD [3]. CVD is a complex group of disorders, which involves peripheral arterial disease (PAD), coronary artery disease (CAD), cerebrovascular disease (CBD) and rheumatic heart disease (RHD) [1,2,3,4]. According to the World Health Organization (WHO), CVD is one of the leading causes of death globally, estimated to cause more than 17.9 million casualties each year [5]. The modern way of life such as daily diet, increased stress accompanied with high working hours and lack of physical exercise are the major factors that are related to the increased CVD occurrence [6,7]. Moreover, the proper management of CVD still represents a great burden from an economical point of view and on national health care systems, which are also characterized by major deficiencies in their daily routine [8,9].

The primary reason for CVD development is atherogenesis and developing atherosclerosis in the patient’s vascular network [3,10]. In addition, an individual’s genetic background plays a crucial role in disease progression. Therefore, the understanding of the underlying, associated pathogenetic factors may provide further insight for the better administration of CVD.

Currently, advanced therapeutic strategies are utilized in CVD patients, including the use of pharmaceutical regimens and modern vascular graft bioengineering applications [3]. To date, the gold standard procedure for the replacement of the occluded coronary arteries relies on the use of autologous secondary vessels such as the internal thoracic artery and saphenous vein [11]. In addition, fabricated vascular grafts made of synthetic materials such as Dacron and expanded polytetrafluoroethylene (ePTFE) have been applied in patients, although the success rate is variable [12]. Intriguingly, significant adverse reactions have occurred regarding the use of both strategies. Moreover, suitable autologous vascular grafts can be found in less than 30% of CVD patients, while synthetic SDVGs are characterized by a reduced patency rate (<60%) within the first year of application [3]. Other common manifestations include neointima formation and vessel occlusion, initiation of the calcification process and a severe host immune reaction [3]. These manifestations could lead to new vascular graft transplantation, which represents an unfavorable situation for the majority of the patients. Importantly, the application of synthetic SDVGs in pediatric patients is prohibited, due to their poor biological properties such as limited size alteration [3].

Therefore, and in order to avoid the aforementioned side effects, modern approaches in CVD must be evaluated as alternative therapeutic strategies. In this way, the application of advanced tissue engineering methods such as the decellularization protocol is currently being investigated for the potential SDVGs manufacturing [13,14,15]. The application of the decellularization method for the efficient production of SDVGs has gained attraction from the scientific community over the last decade [3]. Decellularization aims to produce an acellular biological scaffold through the removal of the tissue-resident cellular populations, while at the same time preserving adequately the extracellular matrix (ECM) [13,14,15]. The choice of the decellularization protocol is dependent on the tissue’s origin and is necessary in order to produce a well-defined acellular scaffold. Utilizing the decellularization procedure, biological scaffolds can be efficiently derived from large animal models (such as porcine and bovine animal models), cadaveric donors, or discarded biological materials [13,14,15]. Besides the proper scaffold production, recellularization with host cellular populations must be performed in order to reduce any adverse reactions incidence. Taking into consideration the above, human umbilical arteries (hUAs) may represent a valuable alternative source for the production of functional SDVGs [16]. HUAs can be noninvasively isolated after gestation from the umbilical cord. Their inner lumen diameter ranges between 1–6 mm, and are characterized by three different wall layers (tunica intima, media and adventitia) and hence could resemble the structural function of human coronary arteries [16]. Additionally, animal-derived and biohybrid-fabricated vessels have been proposed [3]. However, the persistence of α-gal epitopes in decellularized scaffolds and the low biomechanical properties could limit their off-the-shelf application [17]. Additionally, advanced 3D bioprinting methods could enhance the SDVGs production process [18]. New bioprinting methodologies such as 4D printing will create a new era for the production of the next-generation (shape-shifted) vascular grafts [19]. In the near future, repopulated SDVGs with genetically engineered cellular populations, will be presented and used successfully in CVD patients, efficiently prolonging their lifetime. In the context of advanced approaches in CVD, the current Special Issue of *Bioengineering* aimed to gather modern studies related to translational vascular medicine. The current Special Issue included three original articles, two review articles and one editorial article. 

The first article published by Mallis et al. [20] provided a comprehensive review regarding the fabrication of SDVGs and also the future perspectives, which will accompany their potent application. 

Furthermore, Kozaniti et al. [21] presented the last evidence regarding the 3D printing approach and how can be associated with the production of functional vascular grafts.

Moreover, Garcia-Sabate et al. [20] developed a novel biomimetic 3D model to mimic atherosclerosis in order to investigate thoroughly the role of monocytes and macrophages in disease progression. Their work proved that the collagen density in the atherosclerotic plaque is the driving cause, thus inducing the secretion of specific adipokines and growth factors from the macrophages. The macrophage modulation mediated by the collagen density is further related to the atherosclerosis disease progression [22]. 

This Special Issue also involved an additional original research article prepared by Mallis et al. [23] More specifically, this study provided evidence regarding the improvement of the repopulation methodology utilizing the cord blood platelet lysate (CBPL). In this study, the Mesenchymal Stromal Cells (MSCs) were dynamically seeded on decellularized vascular grafts. The performed histological analysis indicated the efficient repopulation of the vascular grafts with the MSCs. Indeed, vascular grafts treated with CBPL showed higher MSCs repopulation efficacy compared with the control group, as was determined by Ki67 and mitogen-activated protein (MAP)-kinase expression [23]. This proof-of-concept study indicated that the CBPL may improve the repopulation process, which may further reduce the processing time for bioengineered vascular graft production. Global research effort must be focused on the improvement of the functional vascular grafts manufacturing process. We hope that the current Special Issue of *Bioengineering* will motivate and inspire researchers of the field, worldwide. In this way, more data will be gathered, highlighting significant aspects which can be utilized in cardiovascular therapeutics, and in parallel improving the application of these advanced methods in terms of economics and quality.

## Data Availability

Not applicable.
